# Дифференциальная диагностика и тактика ведения пациента с первичным гипофизитом на примере клинического случая

**DOI:** 10.14341/probl13311

**Published:** 2024-01-24

**Authors:** Н. Н. Катамадзе, А. А. Цкаева, Е. А. Пигарова, Л. К. Дзеранова, Н. В. Тарбаева

**Affiliations:** Национальный медицинский исследовательский центр эндокринологии; Национальный медицинский исследовательский центр эндокринологии; Национальный медицинский исследовательский центр эндокринологии; Национальный медицинский исследовательский центр эндокринологии; Национальный медицинский исследовательский центр эндокринологии

**Keywords:** гипофизит, аутоиммунные эндокринопатии

## Abstract

За последние годы наблюдается значительный рост распространенности аутоиммунных эндокринопатий, которые, как известно, поражают различные органы эндокринной системы, включая гипофиз. Гипофизит — это общий термин, используемый для описания любой формы селлярного и супраселлярного воспаления, которое приводит к структурным изменениям в гипоталамо-гипофизарной области и проявляется различной степенью гипопитуитаризма. На сегодняшний день выделяют первичный — возникает вследствие аутоиммунного поражения непосредственно гипофиза и вторичный — вследствие наличия системного аутоиммунного заболевания — гипофизит. Независимо от этиологии у пациентов с гипофизитом возникают различные признаки и симптомы, вызванные воспалительным процессом в области гипофиза. МРТ в настоящее время является лучшим неинвазивным инструментальным методом для диагностики гипопитуитаризма, однако подтвердить диагноз можно только по данным гистологического исследования ткани гипофиза, что требует оперативного вмешательства, которое не всегда целесообразно. В представляемой статье нами описан пациент с МРТ-признаками гипофизита при отсутствии характерной клинической симптоматики.

## Abstract (en)

In recent years, there has been a significant increase in the prevalence of autoimmune endocrinopathies, which are known to affect various levels of the endocrine system, including the pituitary gland. Hypophysitis is a general term used to describe any form of sellar and suprasellar inflammation that leads to structural changes in the hypothalamic-pituitary region and manifests itself in varying degrees of hormonal deficiency of the anterior and posterior pituitary glands. To date, there is a primary form of hypophysitis, which occurs as a result of an autoimmune lesion directly to the pituitary gland, and a secondary form of hypophysitis, which occurs as a result of the presence of a systemic autoimmune disease. Regardless of the etiology, patients with hypophysitis show various signs and symptoms caused by an inflammatory process in the pituitary gland, which can lead to the development of hypopituitarism, compression of the sellar and parasellar structures. MRI is currently the best non-invasive diagnostic tool for diagnosing hypopituitarism, however, the diagnosis can be made with certainty only by histological examination of the pituitary tissue, which requires an invasive approach, which greatly reduces the feasibility of this procedure. In this article, we present a patient with MRI showing signs of hypophysitis in the absence of clear clinical symptoms.

## ВВЕДЕНИЕ

Гипофизит — это воспалительное заболевание, характеризующееся неопухолевой инфильтрацией гипофиза, приводящее к нарушению его функций и увеличению объема. Распространенность оценивается как 1 случай на 7–9 млн населения в год. На долю гипофизита приходится 0,24–0,88% всех заболеваний гипофиза [1].

На сегодняшний день выделяют первичную форму, которая возникает вследствие аутоиммунного поражения непосредственно гипофиза, и вторичную — вследствие системного вовлечения при таких заболеваниях, как саркоидоз, гемохроматоз, гранулематоз Вегенера, болезнь Такаясу, гистиоцитоз Лангерганса, сифилис, туберкулез — формы гипофизита, гораздо реже причиной развития заболевания может служить применение иммуномодулирующих лекарственных средств (интерферон-α, рибавирин, ингибиторы иммунных контрольных точек — PD-1/PD-L1 и CTLA-4) [1][2].

Лимфоцитарный гипофизит — наиболее распространенная форма гипофизита, имеет аутоиммунный генез и чаще наблюдается у женщин во время беременности или после родов. Гранулематозный гипофизит — второй по частоте вариант гипофизита, чаще всего причина заболевания остается неизвестной. Перед заключением, что гранулематозный гипофизит является идиопатическим, следует исключить возможные вторичные причины гранулематозной инфильтрации гипофиза (например, ­наличие у пациента туберкулеза). Ксантоматозный, некротизирующий и IgG4-ассоциированный гипофизит встречаются очень редко, последний часто является проявлением системного заболевания с полиорганным поражением (IgG4-ассоциированное заболевание) [3–10].

Ингибиторы иммунных контрольных точек представляют собой моноклональные антитела, которые все чаще используются для лечения солидных и гематологических злокачественных новообразований. Они вызывают активацию и пролиферацию Т-лимфоцитов, которые приводят к противоопухолевому ответу и могут вызывать аутоиммунные проявления, известные как часть так называемых «иммуноопосредованных нежелательных явлений». У значительного числа пациентов, получающих ингибиторы иммунных контрольных точек, развивается иммуноассоциированный гипофизит, требующий своевременной диагностики и лечения [11].

Независимо от этиологии у пациентов с гипофизитом проявляются различные признаки и симптомы, вызванные воспалительным процессом в области гипофиза, которые могут привести к развитию гипопитуитаризма, сдавлению турецкого седла и параселлярных структур.

Клиническая картина гипофизита варьирует в зависимости от распространенности воспалительного процесса. При поражении аденогипофиза ожидаются различные степени гипопитуитаризма и симптомы компрессии — головная боль и/или нарушения остроты зрения. При вовлечении задней доли или воронки гипофиза наиболее частым симптомом является резистентность к антидиуретическому гормону (АДГ) (ранее несахарный диабет). Гипопитуитаризм в исходе гипофизита имеет свои особенности. В отличие от других причин гипопитуитаризма, с дефицитом АКТГ (адренокортикотропного гормона) и ТТГ (тиреотропного гормона) можно столкнуться на ранних стадиях заболевания [12][13].

Диагноз гипофизита ставится на основании клинических, лабораторных и радиологических данных. Несмотря на то что биопсия гипофиза является золотым стандартом диагностики первичного гипофизита, ее следует использовать только в исключительных случаях. Магнитно-резонансная томография (МРТ) является методом выбора при дифференциальной диагностике гипофизита с аденомами гипофиза у взрослых, герминомой и лангергансоклеточным гистиоцитозом у подростков, а также метастазами у лиц, получающих ингибиторы иммунных контрольных точек [2].

На сегодняшний день аутоиммунный гипофизит остается диагнозом исключения и может быть с уверенностью поставлен только при гистологическом исследовании ткани гипофиза, что требует инвазивного подхода [14][15].

Основой лечения пациентов с гипофизитом является компенсация гипофизарной недостаточности. Однако в исключительных случаях, при возникновении симптомов компрессии и риска потери зрения, применяют высокие дозы глюкокортикоидов, что, как правило, дает превосходный первоначальный ответ, хотя и рецидивы являются частым явлением [16].

## ОПИСАНИЕ СЛУЧАЯ

Пациент Б., 54 лет поступил в отделение нейроэндокринологии ФГБУ «НМИЦ эндокринологии» Минздрава России с жалобами на общую слабость, непостоянные односторонние головные боли пульсирующего характера, учащенное мочеиспускание в ночное время (3–4 раза за ночь), сухость во рту, периодическую жажду (4–5 л в сутки). Во время госпитализации диагностирован гипопитуитаризм вследствие инфильтративного образования гипофиза (гипофизит): вторичный гипотиреоз, вторичный гипогонадизм. С целью уточнения наличия дефицита АДГ выполнена проба с водной депривацией. В ходе пробы на фоне дегидратации гипернатриемии и повышения осмоляльности плазмы не выявлено (табл. 1), максимальный уровень натрия крови — 144 ммоль/л, осмоляльность крови — 298 мОсм/кг, максимальная осмоляльность мочи — 449 мОсм/кг, также проведена инфузионная проба с гипертоническим (3%) раствором NaCl (табл. 2), в ходе которой максимальный уровень натрия крови — 144 ммоль/л, осмоляльность крови — 303 мОсм/кг, максимальная осмоляльность мочи — 428 мОсм/кг. Убедительных данных за наличие дефицита АДГ не получено. Пациенту выполнена проба с 0,1 мг десмопрессина (табл. 3), по результатам которой осмоляльность мочи через 2 ч — 491 мОсм/кг, через 4 ч — 586 мОсм/кг, что расценено как нефрогенно-опосредованное снижение концентрационной способности почек без тенденции к обезвоживанию, с целью избегания водной интоксикации прием десмопрессина прекращен, без ухудшения самочувствия.

**Table:** Таблица 1. Результаты теста с водной депривацией

Время	Вес, кг	Объем мочи, мл	АД/Ps	Самочувствие	Натрий сыворотки крови, ммоль/л	Osm плазмы, mOsm/кг	Osm мочи, mOsm/кг
08:30	87,4	200	110/70 (72)	Сухость во рту	142,8	297	370
09:30	87,2	100	110/70 (74)	Жажда, сухость во рту	-	-	433
10:30	87,15	20	120/80 (70)	Жажда, сухость во рту	-	-	476
11:30	87,15	100	110/75 (70)	Жажда, сухость во рту	143	297	432
12:30	86,85	100	100/70 (72)	Жажда, сухость во рту	-	-	430
13:30	86,55	40	110/70 (73)	Жажда, сухость во рту	144,8	298	449

**Table:** Таблица 2. Результаты пробы с гипертоническим раствором

Время	АД/Ps	Самочувствие	Натрий сыворотки крови, ммоль/л	Osm плазмы, mOsm/кг	Osm мочи, mOsm/кг
09:30	110/75 (72)	удовлетворительное	141,3	291	316
11:15	110/80 (70)	удовлетворительное	138,8	293	-
11:45	115/70 (70)	удовлетворительное	142	297	-
12:15	115/80 (71)	удовлетворительное	141,6	296	399
12:45	125/80 (72)	удовлетворительное	141,9	297	316
13:15	125/80 (70)	удовлетворительное	143,2	299	428
13:45	125/80 (70)	удовлетворительное	144	303	-

**Table:** Таблица 3. Протокол теста с 0,1 мг десмопрессина

Время	Вес, кг	Объем мочи, мл	АД/Ps	Самочувствие	Osm мочи, mOsm/кг
15:45	87,0	-	110/80 (70)	удовлетворительное	491
17:45	87,0	-	115/70 (72)	удовлетворительное	586

В отделении подтвержден вторичный гипотиреоз (Т4 св. 9,0 пмоль/л (9–19 пмоль/л), ТТГ 0,051 мМЕ/л (0,2–4 мМЕ/мл)), инициирована терапия левотироксином натрия 100 мкг/сут, выявлен гипогонадотропный гипогонадизм (общий тестостерон 0,373 нмоль/л, лютеинизирующий гормон 0,216 Ед/л), однако от терапии препаратами тестостерона решено воздержаться вследствие невозможности на данном этапе полностью исключить герминому как причину инфильтрации гипофиза. Данных за наличие вторичной надпочечниковой недостаточности не выявлено (утренний кортизол крови — 614 нмоль/л и отсутствие клинических проявлений). Клинический и биохимический анализы крови без значимых изменений.

МРТ головного мозга показала гиперплазию, деформацию и увеличение размеров гипофиза, отсутствие типичного сигнала от задней доли гипофиза, нарушение функций нейрогипофиза более вероятно соответствуют гипофизиту, проведение контрастного усиления не выявило каких-либо дополнительных изменений гипоталамо-гипофизарной области.

Проведен поиск возможных причин вторичного гипофизита: проведена мультиспиральная компьютерная томография органов грудной и брюшной полости с контрастным усилением: КТ-картина единичных очагов в обоих легких, вероятно, поствоспалительного характера, сегментарной гиперплазии левого надпочечника, дивертикулеза сигмовидной кишки, атеросклероза аорты и ее ветвей. Таким образом, убедительных данных за наличие саркоидоза, гистиоцитоза, туберкулеза и метастатического поражения гипофиза не получено.

Выполнено УЗИ щитовидной железы, согласно которому выявлены эхографические признаки многоузлового зоба (EU-TIRADS 2). Пациент консультирован офтальмологом, согласно заключению, выявлена ангиопатия сетчатки обоих глаз.

Все показатели биохимического анализа крови пациента без клинически значимых изменений (табл. 4).

**Table:** Таблица 4. Результаты биохимического анализа крови

Показатели	Значение	Ед. измерения	Референсный интервал
Холестерин общий	4,77	ммоль/л	3,3–5,2
Холестерин ЛПНП	2,47	ммоль/л	1,1–3
Холестерин ЛПВП	1,413	ммоль/л	0,9–2,6
Триглицериды	1,98	ммоль/л	0,1–1,7
Креатинин	78,9	мкмоль/л	63–110
Мочевина	3,13	ммоль/л	3,2–7,4
Мочевая кислота	342,02	мкмоль/л	202–416
АЛТ	17,5	Ед/л	0–55
АСТ	15,6	Ед/л	5–34
Билирубин общий	9,2	мкмоль/л	3,4–20,5
Белок общий	72,1	г/л	64–83
Кальций общий	2,34	ммоль/л	2,15–2,55
Альбумин	42,5	г/л	35–50
Осмоляльность плазмы	294	mOsm/кг	280–300
Натрий	141,5	ммоль/л	136–145
Хлориды	107,1	ммоль/л	98–107
Калий	4,88	ммоль/л	3,5–5,1

Пациент повторно госпитализирован в ФГБУ «НМИЦ эндокринологии» Минздрава России через 6 мес с целью динамического наблюдения. Согласно данным лабораторного обследования: уровень инсулиноподобного фактора роста 1 типа в пределах референсных значений — 110,9 нг/мл. Вторичный гипотиреоз компенсирован — уровень Т4св. — 14 пмоль/л, коррекции дозы левотироксина натрия не требовалось. Уровень тестостерона — 14,3 нмоль/л, т.е. данных за гипогонадизм не получено.

По результатам МРТ головного мозга отмечалась положительная динамика в отношении воспалительного процесса в гипофизе: деформация и выраженная неоднородность структуры гипофиза, нарушение функций нейрогипофиза, соответствующие гипофизиту, по сравнению с предыдущим исследованием — уменьшение вертикального размера гипофиза.

## ОБСУЖДЕНИЕ

Аденомы гипофиза являются наиболее частыми новообразованиями головного мозга с распространенностью в популяции 0,1% [17]. Приблизительно 65% аденом гипофиза гормонпродуцирующие: 48% составляют пролактиномы, 10% — соматотропиномы, 6% — кортикотропиномы и менее 1% — тиреотропиномы [18].

Остальные 35% составляют гормонально-неактивные аденомы гипофиза. Аденомы, не имеющие гормональной активности, проявляются неврологическими симптомами, хиазмальным синдромом, головной болью или гипофизарной недостаточностью вследствие сдавления объемом опухоли окружающих структур [19][20] или являются случайной находкой при лучевых методах исследования. В данном контексте важно понимать, что существуют и другие неопухолевые причины нарушения функций гипофиза, для которых не всегда показано хирургическое лечение. Гипофизит следует рассматривать как одну из патологий в данной категории.

Воспалительный процесс может поражать как переднюю, так и заднюю доли гипофиза. Следовательно, в соответствии с анатомическим строением железы выделяют аденогипофизит (воспалительный процесс в передней доле гипофиза), инфундибулонейрогипофизит (воспаление воронки и задней доли гипофиза), пангипофизит (поражение всей железы) (рис. 1) [21][22].

**Figure:**
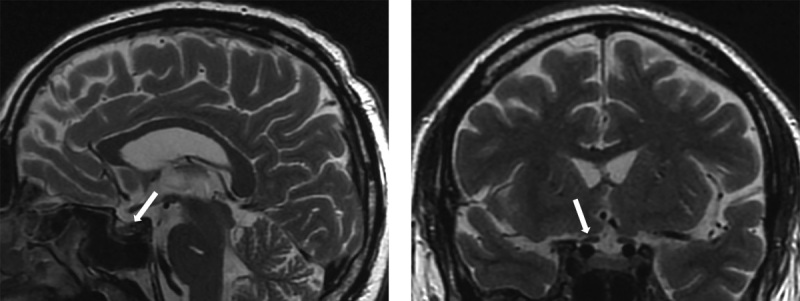
Рисунок 1. МР-картина хиазмально-селлярной области. Сагиттальный и фронтальный срезы.

Согласно данным морфологических исследований, сформированы основные современные принципы классификации гипофизитов: лимфоцитарный (самая частая форма, составляет около 68%) — при наличии скоплений CD4+-T-хелперов и В-лимфоцитов; гранулематозный — при наличии скоплений эпителиоидных гистиоцитов и многоядерных гигантских клеток; ксантоматозный — при наличии богатых липидами пенистых гистиоцитов и некротический — при наличии иммунного инфильтрата в некротизированной ткани гипофиза [3–10][23–25]. Кроме того, в литературе описаны смешанные формы гипофизитов.

В последние годы выделяют IgG4-ассоциированный гипофизит (4%), характеризующийся наличием мононуклеарной инфильтрации лимфоцитами и плазматическими клетками [26]. Гипофизит, связанный с IgG4, также был связан с воспалительным поражением многих других органов, таких как поджелудочная железа, желчевыводящие пути, забрюшинное пространство, средостение, щитовидная железа, мочеполовые пути, слюнные и слезные железы, орбиты [27].

В настоящее время биопсия гипофиза не проводится рутинно, что обусловлено высокой частотой недостаточного объема материала для постановки диагноза и развития разной степени гипопитуитаризма, поэтому клиническое применение гистологической классификации существенно ограничено. Фактически гипофизит классифицируется в соответствии с распространением воспалительного процесса и наличием внегипофизарных изменений.

По этиологии гипофизит определяется как первичный, если воспаление затрагивает непосредственно ткань гипофиза и имеет аутоиммунную природу, тогда как вторичный гипофизит является следствием системных аутоиммунных [27–29] или инфекционных заболеваний [30][31], а также лечения иммуномодулирующими препаратами (интерферон-α, рибавирин или ингибиторы иммунных контрольных точек) [32–34]. Случаи вторичного гипофизита также ассоциированы с разрывом кисты кармана Ратке (при морфологическом исследовании может проявляться как лимфоцитарная, ­гранулематозная, ксантоматозная или смешанная формы гипофизита), краниофарингиомой, лимфомой гипофиза [35–40].

Несмотря на то что аутоиммунная природа первичного гипофизита хорошо известна, патогенные аутоантигены, являющиеся причиной данного заболевания, не идентифицированы. Серологические тесты, основанные на исследовании специфических аутоантител в крови, в настоящее время не разработаны [35].

Диагностика гипофизита в большинстве случаев осуществляется по клиническим критериям [41], так как морфологический анализ ткани гипофиза проводится редко. Данное исследование может применяться при наличии показаний к нейрохирургическому вмешательству с целью устранения симптомов сдавления [42][43]. Нередко диагноз гипофизита ставится на основании значимого уменьшения размеров объемного образования по данным МРТ без какого-либо предшествующего лечения.

Гипофизит характеризуется широким спектром клинических проявлений: от бессимптомного течения до наличия одного или нескольких признаков гипопитуи­таризма, острой манифестации, включая гипофизарную апоплексию и даже смерть от циркуляторного коллапса или надпочечникового криза.

Гипофизит часто мимикрирует под гораздо более распространенную патологию хиазмально-селлярной области — гормонально-неактивную макроаденому гипофиза. Приблизительно 40% пациентов с аутоиммунным гипофизитом ошибочно диагностируются и подвергаются ненужному хирургическому вмешательству [52].

МРТ в настоящее время является лучшим неинвазивным диагностическим инструментальным методом для дифференциации аутоиммунного гипофизита от несекретирующих аденом [53].

В 2009 г. было предложено систематизировать диагностические критерии согласно данным МРТ с целью более точно дифференцировать гипофизит и аденомы гипофиза и, как следствие, избежать неоправданного хирургического вмешательства. В результате авторами исследования были предложены 8 основных критериев, позволяющих провести дифференциальную диагностику. К ним относились возраст пациента, наличие беременности, размеры гипофиза, характер и скорость накопления контрастного препарата, симметричность увеличения гипофиза, визуализация нейрогипофиза, увеличение размеров воронки гипофиза, изменение слизистой оболочки в пазухах основной кости и/или прилегающих ячейках решетчатой кости. В результате была предложена балльная система оценки показателей МРТ, которая не нашла широкого применения в клинической практике [2].

Gutenberg A. и соавт. предлагают использовать следующие МР-критерии гипофизита: увеличение объема железы, утолщение ее воронки, неоднородность сигнала от ткани аденогипофиза и кистозные изменения передней доли гипофиза, характерное накопление контраста менингеальными структурами («дуральный хвост») на сагиттальном срезе, отсутствие гиперинтенсивного сигнала на Т1-взвешенных изображениях [52].

С целью визуализации хиазмально-селлярной области нашему пациенту была выполнена МРТ.

Согласно данным исследования у пациента выявлены типичные МР-признаки гипофизита: синдром «дурального хвоста», увеличение объема гипофиза, диффузная неоднородность сигнала от ткани аденогипофиза.

МРТ-исследование не выявило опухолевых образований, однако визуализированный гипофиз деформирован, его размеры составили по вертикальному сечению — 8 мм, поперечному — 16 мм, переднезаднему — 15 мм (рис. 1). Тогда как размеры гипофиза при предыдущем исследовании были больше — 13×22×16 мм соответственно (рис. 2). Структура аденогипофиза неоднородна, в том числе за счет формирования фиброзных изменений в задней части, а при контрастном усилении отмечается выраженное неоднородное накопление контрастного вещества с наличием гиповаскулярных участков, соответствующих фиброзным изменениям. Выявлено активное накопление парамагнетика прилегающей твердой мозговой оболочкой, что соответствует синдрому «дурального хвоста». Воронка не утолщена, расположена срединно. Задняя доля гипофиза достоверно не визуализировалась.

**Figure:**
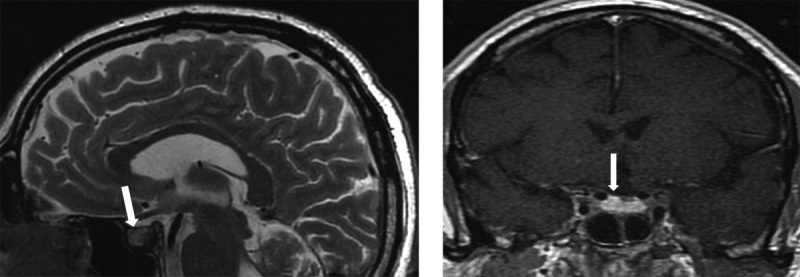
Рисунок 2. МР-картина хиазмально-селлярной области. Сагиттальный и фронтальный срезы.

С целью оценки наличия гормональной недостаточности передней и/или задней долей гипофиза исследуют широкий спектр гормонов: кортизол, АКТГ, инсулиноподобный фактор роста-1, эстрадиол (для женщин до менопаузы)/тестостерон, лютеинизирующий, фолликулостимулирующий, тиреотропный гормоны, свободный Т4, пролактин, осмоляльность плазмы/мочи, электролиты. В то время как исследование рутинных показателей ­крови (общеклинический анализ крови, почечный, печеночный профиль, маркеры костного обмена, С-реактивный белок, скорость оседания эритроцитов) могут пролить свет на понимание природы системного процесса [23].

Диагностика гипофизита требует тщательной оценки других возможных неопластических поражений, инфильтративных заболеваний, инфекций и системных воспалительных процессов.

При подозрении на наличие таковых рекомендован расширенный поиск с целью уточнения истинной причины гипофизита [23].

Увеличение размеров гипофиза приводит к развитию хиазмально-селлярного синдрома, (68%), частыми клиническими проявлениями данного синдрома являются головные боли и сужение полей зрения [42][44–47].

Нарушение функций гормонпродуцирующих клеток гипофиза воспалительным инфильтратом с дальнейшим уменьшением их количества приводит к развитию гипопитуитаризма. При этом в большинстве случаев возникает характерный для данного заболевания синдром гиперпролактинемии, который развивается в результате снижения ингибиторного влияния дофамина при сдавлении ножки гипофиза [48][49].

Клиническая картина гипофизита включает в себя все виды гипофизарной дисфункции. Надпочечниковая недостаточность, согласно литературным данным, встречается в 8–86% случаев, тогда как центральный гипотиреоз и гипогонадизм — в 10–77 и 1–62% случаев соответственно. Дефицит гормона роста и АДГ наблюдается у 14–62 и 20–83% пациентов соответственно [41][42][44][50].

В данной статье мы представляем пациента с идиопатическим первичным гипофизитом, которому не проводилось серологической диагностики или нейрохирургического лечения (в условиях отсутствия показаний к операции), в то время как все причины вторичного гипофизита были исключены.

Данный клинический случай примечателен тем, что исходно картина гипопитуитаризма вследствие гипофизита у пациента была только частичной, с регрессом некоторых его элементов во времени и при уменьшении размеров гипофиза. Эндокринная манифестация у представляемого нами больного включала дефицит гормонов передней доли гипофиза (центральный гипотиреоз) при сохранной функции задней доли. Также обращает на себя внимание разрешение центрального гипогонадизма за 6 мес наблюдения, что, вероятно, связано с восстановлением функции гонадотрофов в условии снижения воспаления.

Данных о регрессии симптомов гипофизита в современной литературе мало, однако обзор 76 случаев первичного гипофизита в Германии показал улучшение клинических и лабораторных показателей у пациентов с нарушением функции гипофиза в 27% случаев, а регрессия воспалительного процесса гипофиза отмечалась у 46% наблюдаемых [44].

Вместе с тем примечательно, что у пациента при отсутствии гиперинтенсивного сигнала от нейрогипофиза на фоне проведения тестов с осмотической стимуляцией (проба с сухоядением и инфузионная проба с 3% гипертоническим раствором) развитие недостаточности АДГ диагностировано не было.

Принимая во внимание данные литературы, отмечается, что отсутствие типичного гиперинтенсивного сигнала на МРТ от нейрогипофиза является отличительной особенностью гипофизитов любой этиологии. Вероятно, данные изменения связаны с наличием воспалительного процесса в мягких тканях гипофиза, который не дает возможности зарегистрировать свечение фосфолипидных гранул от задней доли гипофиза.

A. Bhansali и соавт. представили серию клинических случаев пациентов с идиопатическим гигантоклеточным гранулематозным гипофизитом. Наиболее частыми симптомами у исследуемых были головная боль и нарушение зрения. У всех пациентов диагностирован гипогонадизм, у четырех — гипофункция коры надпочечников и у трех — гипотиреоз. Симптомов недостаточности АДГ ни у одного из исследуемых не наблюдалось. Однако у всех пациентов отмечалось отсутствие гиперинтенсивного сигнала от задней доли гипофиза [51].

Основными задачами при лечении первичного гипофизита являются компенсация гипофизита, снижение воспалительного процесса в гипофизе и устранение синдрома сдавления увеличенного гипофиза. При вторичном гипофизите необходимо разрешение основного заболевания.

Учитывая отсутствие признаков синдрома сдавления, нами была выбрана консервативная тактика ведения, пациенту назначена заместительная терапия левотироксином натрия, что привело к компенсации заболевания согласно данным лабораторного исследования через 2 мес после диагностики вторичного гипотиреоза.

Консервативная тактика — метод выбора при лечении гипофизита любой этиологии при условии отсутствия прогрессирующего характера хиазмального синдрома. Однако при развитии симптомов компрессии терапией первой линии являются глюкокортикостероиды, что приводит к уменьшению объемов гипофиза и облегчению симптомов сдавления хиазмы в течение нескольких недель. Как правило, начальная доза преднизолона составляет 30–40 мг в день в течение 2–4 нед, затем постепенно снижается в течение 2–6 мес.

Однако необходимо помнить, что рецидивы при лечении гипофизита встречаются довольно часто, и в таком случае необходимы многократные курсы высоких доз глюкокортикоидов [16][54][55].

В случаях резистентности к глюкокортикоидам или при возникновении выраженных побочных эффектов используются иммуносупрессанты, такие как азатиоприн, метотрексат и циклоспорин А. Однако долгосрочные последствия применения данных препаратов недостаточно изучены [56].

Lupi I. и соавт., согласно опубликованному обзору литературы, сообщают об уменьшении массы гипофиза в 84% случаев, улучшении функции передней доли гипофиза в 45% и восстановлении функции задней доли гипофиза в 41% случаев после применения высоких доз глюкокортикоидов с относительно низким риском рецидива (14%) [57].

Однако данные результаты, безусловно, должны быть подтверждены в более крупных проспективных исследованиях.

## ЗАКЛЮЧЕНИЕ

Данный клинический случай представляет пример нетипичного течения гипофизита вследствие особенностей проявлений и течения гипопитуитаризма при наличии всех типичных признаков заболевания по результатам МРТ. Особого внимания заслуживает неподтверждение дефицита АДГ (ранее несахарный диабет) на фоне проведения функциональных проб при отсутствии гиперинтенсивного сигнала от нейрогипофиза по МРТ. За последнее десятилетие накоплен значительный объем информации о патофизиологии, диагностике и лечении гипофизита. Однако многие вопросы, в том числе и МР-критерии заболевания, развитие гипофизарной недостаточности и ее регресс, самостоятельно или на фоне проводимой терапии, остаются предметом для обсуждения и требуют дальнейшего исследования.

## ДОПОЛНИТЕЛЬНАЯ ИНФОРМАЦИЯ

Источники финансирования. Государственное задание 1210301000341-1.

Конфликт интересов. Авторы декларируют отсутствие явных и потенциальных конфликтов интересов, связанных с содержанием настоящей статьи.

Участие авторов. Все авторы одобрили финальную версию статьи перед публикацией, выразили согласие нести ответственность за все аспекты работы, подразумевающую надлежащее изучение и решение вопросов, связанных с точностью или добросовестностью любой части работы.
